# Insights into the Function of the CRM1 Cofactor RanBP3 from the Structure of Its Ran-Binding Domain

**DOI:** 10.1371/journal.pone.0017011

**Published:** 2011-02-25

**Authors:** Karla Langer, Cyril Dian, Vladimir Rybin, Christoph W. Müller, Carlo Petosa

**Affiliations:** 1 Structural and Computational Biology Unit, European Molecular Biology Laboratory, Heidelberg, Germany; 2 Institut de Biologie Structurale Jean-Pierre Ebel, Unité Mixte de Recherche 5075 (Commissariat à L'Energie Atomique et aux Energies Alternatives/Centre National de la Recherche Scientifique/Université Joseph Fourier), Grenoble, France; University of Queensland, Australia

## Abstract

Proteins bearing a leucine-rich nuclear export signal (NES) are exported from the nucleus by the transport factor CRM1, which forms a cooperative ternary complex with the NES-bearing cargo and with the small GTPase Ran. CRM1-mediated export is regulated by RanBP3, a Ran-interacting nuclear protein. Unlike the related proteins RanBP1 and RanBP2, which promote disassembly of the export complex in the cytosol, RanBP3 acts as a CRM1 cofactor, enhancing NES export by stabilizing the export complex in the nucleus. RanBP3 also alters the cargo selectivity of CRM1, promoting recognition of the NES of HIV-1 Rev and of other cargos while deterring recognition of the import adaptor protein Snurportin1. Here we report the crystal structure of the Ran-binding domain (RBD) from RanBP3 and compare it to RBD structures from RanBP1 and RanBP2 in complex with Ran and CRM1. Differences among these structures suggest why RanBP3 binds Ran with unusually low affinity, how RanBP3 modulates the cargo selectivity of CRM1, and why RanBP3 promotes assembly rather than disassembly of the export complex. The comparison of RBD structures thus provides an insight into the functional diversity of Ran-binding proteins.

## Introduction

CRM1/Exportin1, a member of the importin-β/karyopherin-β family of nuclear transport factors, is responsible for exporting many proteins and ribonucleoproteins from the nucleus to the cytosol [1]–[4]. Macromolecular cargos exported by CRM1 are characterized by a leucine-rich nuclear export signal (NES), a short, loosely conserved motif first discovered in HIV-1 Rev and protein kinase A inhibitor (PKI) and subsequently identified in over 75 cellular and viral proteins [5]–[7]. CRM1-mediated export is dependent on the small GTPase Ran, whose nucleotide-bound state is regulated by the Ran GTPase-activating protein (RanGAP) and by the guanine nucleotide exchange factor RCC1 [8], [9]. Because RCC1 is restricted to the nucleus whereas RanGAP is excluded from this compartment, Ran is primarily bound to GTP in the nucleus and to GDP in the cytosol – an asymmetric distribution critical for the directionality of nuclear transport [10].

In the nucleus, CRM1 associates in a cooperative manner with RanGTP and with the NES-bearing cargo to form a ternary CRM1/Ran/cargo complex that translocates through the nuclear pore complex (NPC) and subsequently dissociates in the cytosol. In the absence of Ran, CRM1 has low binding affinity for most NES-bearing cargos. However, CRM1 binds tightly to certain cargos, including proteins with supraphysiological NES motifs [11], [12] and Snurportin1 (Spn1), an import adaptor for m_3_G-capped U snRNPs [13]. The crystal structures of human CRM1 in binary complex with Spn1 and in ternary complex with Ran and Spn1 revealed that CRM1 consists of 20 tandem HEAT repeats [14], [15]. These approximately 50-residue motifs comprise two anti-parallel helices (designated A and B) that pack against each other and against neighbouring repeats to form an elongated solenoid [16]. In CRM1, the solenoid adopts a ring-like shape, with the A and B helices defining the outer and inner surfaces, respectively. Ran binds inside the ring, engaging the B helices of N- and C-terminal HEAT repeats as well as a large loop within HEAT repeat 9 [15]. Spn1 binds to the outer surface of CRM1 through an extensive interface involving HEAT repeats 11–16 and three regions of Spn1: the N-terminal NES motif, the nucleotide-binding domain and a C-terminal epitope. The NES motif adopts a helical structure and occupies a hydrophobic groove formed by the A helices of HEAT repeats 11 and 12 [14], [15]. Details of cargo recognition have been further elucidated by structures of CRM1 bound to PKI- and Rev-type NESs [17].

RanBP3 is a Ran-interacting protein with diverse roles in nuclear transport. For example, RanBP3 associates with RCC1 and enhances its catalytic activity towards Ran [18], [19], plays an important role in linking the Ras/ERK/RSK and PI3K/Akt signalling pathways to nuclear transport [20], and stimulates the CRM1-independent nuclear export of β-catenin, Smad2 and Smad3 [21], [22]. However, RanBP3 is best known for its role as a co-factor of CRM1-mediated export. Specifically, RanBP3 enhances the rate of NES export by increasing the affinity of CRM1 for RanGTP, thereby stabilizing the ternary CRM1/Ran/cargo complex in the nucleus [23], [24]. RanBP3 further promotes export complex assembly by increasing the nucleoplasmic pool of CRM1 [25] and by recruiting CRM1 to RCC1, where it facilitates the association of CRM1 with RanGTP [19]. In the absence of Ran and cargo, RanBP3 inhibits CRM1 from interacting with the NPC, thereby reducing futile cycles of transport [24]. Finally, RanBP3 modulates the substrate selectivity of CRM1, enhancing its affinity for the HIV-1 Rev NES while decreasing that for Spn1 [23].

**Figure 1:**
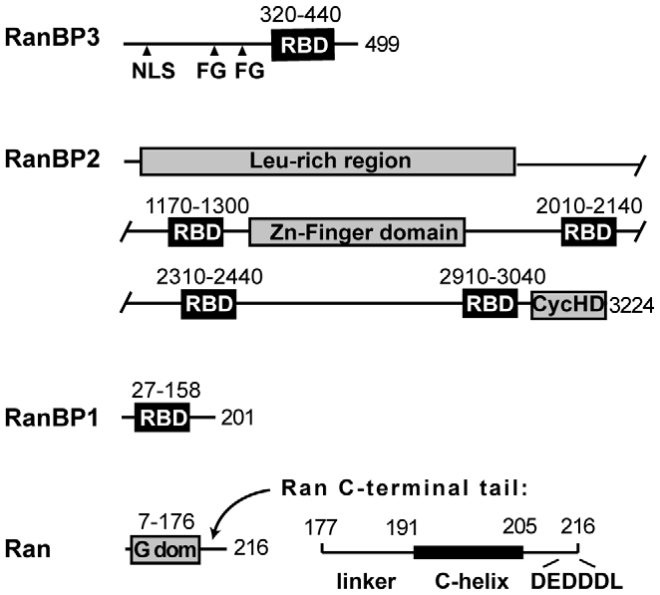
Domain organization of Ran and of Ran-binding proteins. Boundaries of the RBD domains and of Ran elements are indicated. FG, FxFG motif; NLS, nuclear localization signal; CycHD, cyclophilin homology domain; Gdom, G-domain. For clarity, the FG repeats (over 20) of RanBP2 are not shown.

RanBP3 belongs to a class of Ran-binding proteins characterized by one or more Ran-binding domains (RBDs) of approximately 120 residues [18]. The RBD of RanBP3 is flanked by N- and C-terminal regions that are predicted to be intrinsically disordered (Figure 1). The N-terminal region contains a nuclear localization signal (NLS) that is preferentially recognized by importin α3 and is responsible for concentrating RanBP3 in the nucleus [26]. The N-terminal region is also required for RanBP3 to bind CRM1, an interaction putatively mediated by two FxFG motifs located between the NLS and the RBD [24]. Other RBD-containing proteins include RanBP1, a cytosolic protein that comprises little more than a single RBD [27], [28], and RanBP2/Nup358, a giant nucleoporin that localizes to the cytosolic face of the NPC and contains four RBDs [29] (Figure 1). RanBP1 and RanBP2 act as cofactors for RanGAP, enhancing the rate of GTP hydrolysis on Ran by approximately an order of magnitude [30]. RanBP1 and RanBP2 also promote the disassembly of trimeric CRM1/RanGTP/NES complexes in the cytosol [31]–[33]. This activity has been rationalized by the recent crystal structure of yeast CRM1 bound to RanGTP and RanBP1: RanBP1 induces a conformational change in the Ran-interacting HEAT-9 loop of CRM1, leading to constriction of the NES-binding groove and consequent release of the NES [33]. RanBP1 also promotes dissociation of CRM1 from nucleoporins located on the cytoplasmic periphery of the NPC [12], [31], [34], allowing CRM1 to recycle to the nucleus for a new round of export.

Broadly speaking, all three of the above Ran-binding proteins render CRM1-mediated export more efficient. However, whereas RanBP1 and RanBP2 accelerate the terminal steps of export by disassembling the export complex in the cytosol, RanBP3 enhances the initial stages of export by promoting assembly of the CRM1/Ran/NES complex in the nucleus. Another difference is that RanBP1 and RanBP2 bind tightly to RanGTP (with K_d_ values for individual RBDs between 1.2 and 20.8 nM), whereas RanBP3 binds relatively poorly (K_d_∼10 µM) [18], [35], [36]. This presumably allows Ran-bound RanBP3 in the context of a RanBP3/CRM1/Ran/NES complex to be efficiently displaced by RanBP1 or by RanBP2 upon cytosolic entry of the export complex.

To better understand the divergence in function among RBD-containing proteins, we undertook a structural study of the RanBP3 RBD. We determined the high-resolution crystal structure of this domain and compared it to known RBD structures from RanBP1 and RanBP2, previously determined in complex with various binding partners. The analysis sheds light on RanBP3 with regard to its weak Ran-binding activity, its influence on the cargo-binding selectivity of CRM1, and its ability to stabilize a CRM1/Ran/NES complex in the nucleus.

## Results and Discussion

### Structure of the RBD of RanBP3

**Figure 2:**
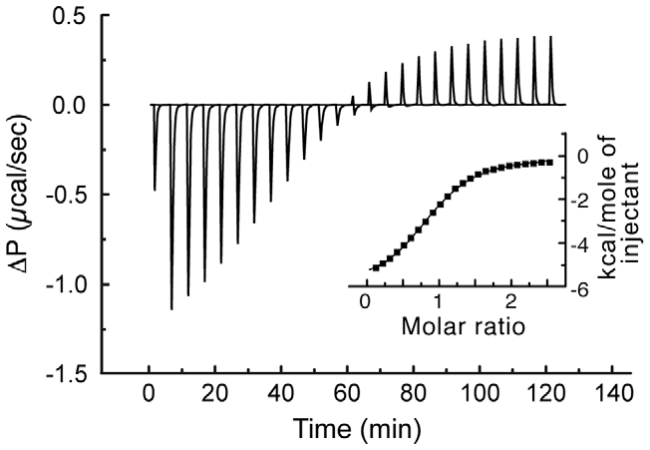
ITC profile of Ran binding by RanBP3 RBD. Differential power (ΔP) time course of raw injection heats for a titration of 800 µM RanBP3 RBD (residues 310–454) into 70 µM RanQ69L∶GTP. The binding reaction and the heat of dilution have different signs. RanQ69L is a mutant form of Ran compromised for intrinsic GTPase activity [8], used to stabilize the active GTP-bound form of Ran over the time course of the experiment. The inset shows normalized binding enthalpies corrected for the heat of dilution as a function of binding site saturation. The solid line represents a nonlinear least squares fit using a single-site binding model. K_d,obs_ was 14±0.3 µM and the stoichiometry was 0.93±0.01.

**Figure 3:**
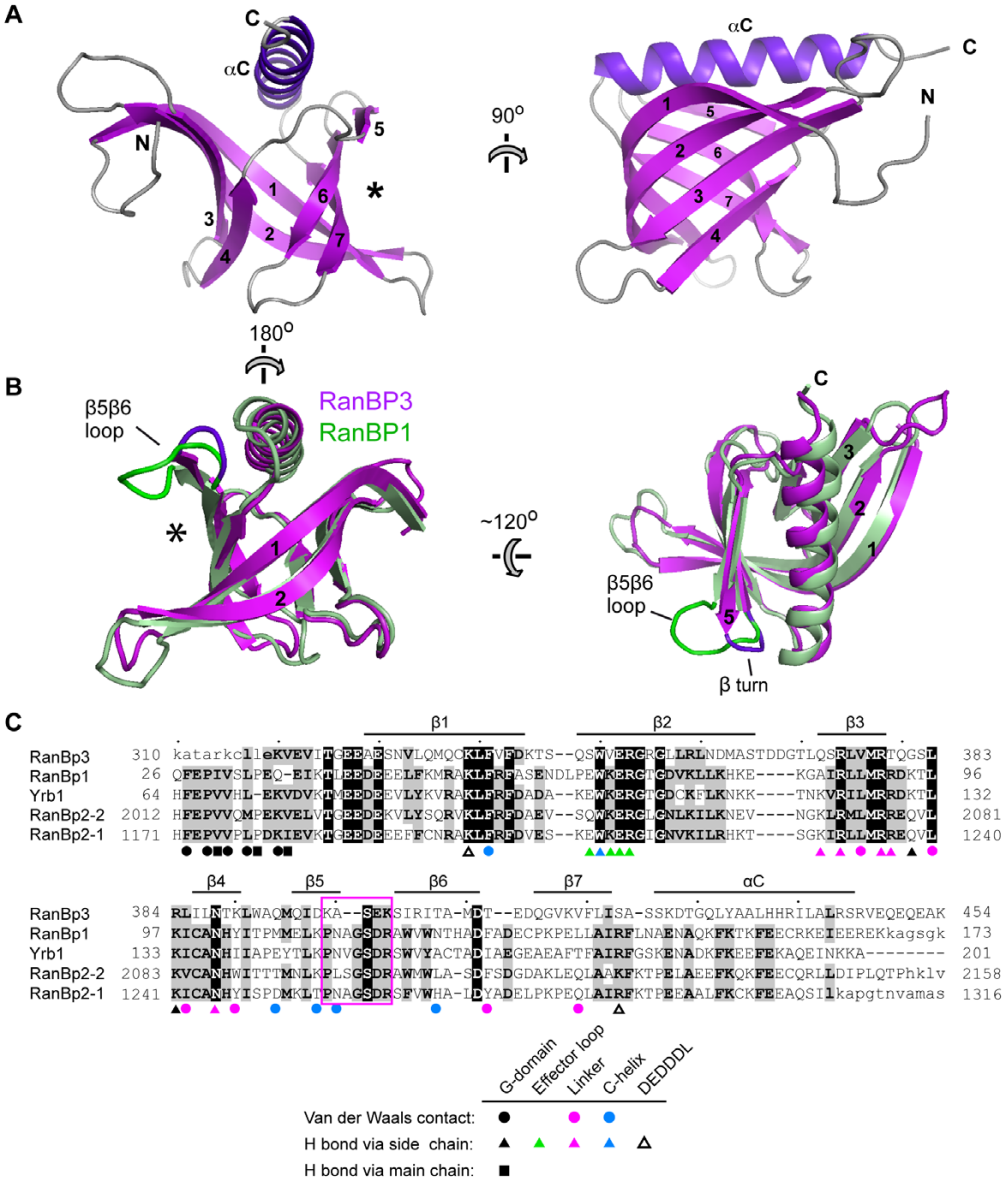
Structure of the RanBP3 RBD. **A**. Ribbon diagram. The asterisk indicates the surface depression which in homologous RBD structures accommodates the Ran C-helix. **B**. Structural alignment of the RanBP3 RBD (magenta) with RanBP1 (green). **C**. Sequence alignment of RBDs of known structure. The β5β6 loop is boxed. Residues in lower case are missing from the structures. RanBP2-1 residues that contact Ran are marked by a circle, triangle or square according to the type of contact (van der Waals, H-bond mediated by a side chain, or H-bond mediated by backbone, respectively) [38]. Marks are coloured according to whether the Ran residue contacted lies in the G-domain (black), effector loop (green), linker (magenta), C-helix (blue) or DEDDDL motif (open triangle).

Initial efforts to crystallize the RBD of RanBP3 in complex with Ran were hampered by the weak Ran-binding affinity of this domain, which we estimated by isothermal titration calorimetry (ITC) to correspond to a K_d_ of 14±0.3 µM (Figure 2; Figure S1 shows the corresponding experiment for full-length RanBP3), in agreement with a previous semi-quantitative study [18]. We therefore pursued the structure of the RBD in its unbound form. We solved the structure at 1.61 Å resolution using experimental phases obtained from a platinum derivative, and at 2.1 Å in a second crystal form by molecular replacement. (Crystallographic statistics are summarized in Table 1). As expected, the RanBP3 RBD adopts a pleckstrin homology fold, composed of 7 anti-parallel β-strands and a C-terminal α-helix. The strands define a continuous sheet with simple up-down topology, forming an imperfect β barrel that juxtaposes strands 4 and 6 and extrudes strand 5 (Figure 3A). The C-terminal helix caps the barrel, packing against strands 1, 2, 5 and 6. The loops at the base of the barrel and a shallow depression on the protein surface between the β1β2 and β5β6 loops (asterisk in Figure 3A and B) correspond to important Ran-binding epitopes in known structures of Ran/RBD complexes. Crystal forms 1 and 2 contain two and four molecules per asymmetric unit, respectively, and aligning these structures reveals variations in the N- and C-terminal residues and in several loops, reflecting the inherent flexibility of these regions (Figure S2; Table S1). In contrast, the β5β6 loop, whose functional role is evoked below, is highly uniform in structure, suggesting a comparatively rigid element. Our crystal structure of the RanBP3 RBD is consistent with an NMR structure determined by a structural genomics consortium [37] (PDB code 2CRF), although aligning the two structures yields a high rmsd value (1.6 Å for 100 Cα residues, omitting variable regions), which we attribute to coordinate errors in the NMR model.

**Table 1:** Crystallographic data collection and refinement statistics.

	Form 1	Form 2
**Data collection:**		
Space group	P3_1_21	P2_1_2_1_2_1_
Unit cell dimensions (Å)	*a* = *b* = 61.3, *c* = 137.2	*a* = 62.9, *b* = 73.4, *c* = 120.4
Resolution range (Å)	35-1.61 (1.70 - 1.61)	48-2.1 (2.21 - 2.1)
ESRF Beamline	ID23-1	ID14-4
Wavelength (Å)	1.0723	0.9795
No. unique reflections	37445 (4741)	33296 (4766)
R_symm_ (%)	5.6 (53.4)	6.3 (38.4)
I/σ(I)	26.7 (4.3)	19.8 (4.8)
Completeness (%)	94.7 (81.1)	100 (100)
Multiplicity	10.7 (9.2)	5.6 (5.7)
**Refinement:**		
Monomers /ASU	2	4
No. protein atoms	1864	3789
No. water molecules	299	246
R_work_/R_free_	0.147 / 0.196	0.193 / 0.225
Rmsd bonds (Å)	0.005	0.003
Rmsd angles (°)	0.890	0.756
*Ramachandran Plot:*		
Most favoured (%)	94.5	93.3
Allowed (%)	5.5	6.5
Generously allowed (%)	0	0.2
Outliers	0	0
Molprobity Score	1.67 (77^th^ percentile)	2.11 (73^rd^ percentile)

Structures are known for human and yeast RanBP1 and for the first and second RBD domains of RanBP2 (RanBP2-1 and RanBP2-2) [33], [38]–[40], all of which bind Ran with high affinity. These domains share less than 25% sequence identity with the RanBP3 RBD and when structurally aligned with our crystal structure yield rmsd_100_ values [41] of 1.6–2.1 Å (Table S2). This reflects much greater divergence than is observed among high-affinity RBDs (e.g., for RanBP1 versus RanBP2-1 the identity is 62% and rmsd_100_ is 0.89 Å). The most notable differences are the positioning of the C-terminal helix relative to the β barrel and the conformation of four loop regions (Figure 3B and Figure S3). In particular, the β5β6 loop in RanBP3 (res. 401–404) is two residues shorter than in the high-affinity RBDs, forming a type I β turn rather than a loop. The high affinity RBDs all possess a bulky aromatic residue (Trp120 in RanBP1) at the base of this loop, causing the latter to project outwards as a prominent protrusion on the protein surface (Figure S4A and B). In contrast, RanBP3 has a smaller (Ile405) residue here, which together with the shortened loop results in a smoother profile. As a result, the RanBP3 exhibits a surface depression that is markedly less pronounced than in the high-affinity RBDs (Figure S4B), which recognize the Ran C-helix through this feature.

### Structural basis of weak Ran binding

**Figure 4:**
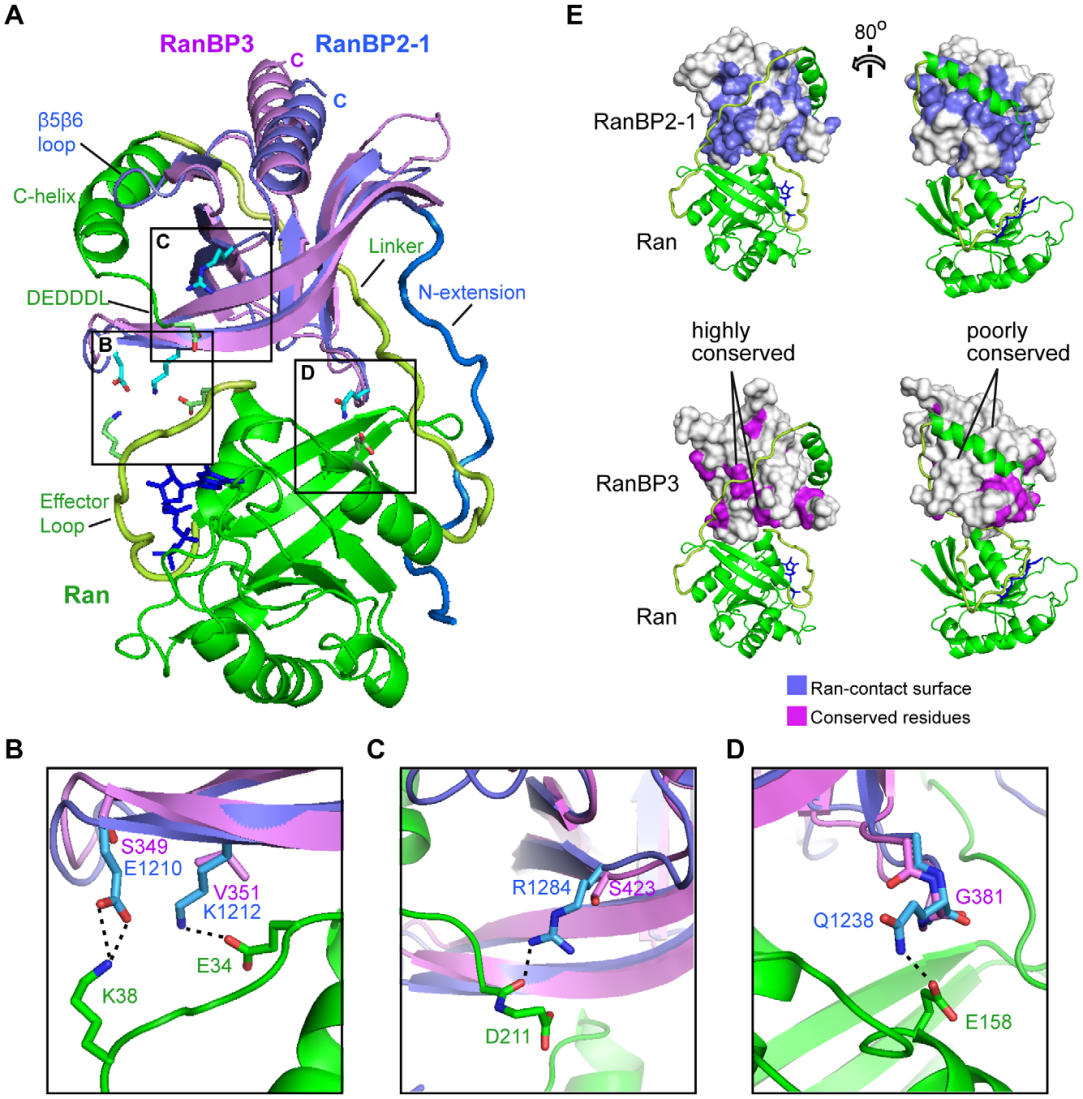
Structural basis of weak Ran-binding. **A**. Structural alignment of the RanBP3 RBD with the RanBP2-RBD1/Ran complex. Ribbon diagrams show Ran (green), RanBP2-1 (blue) and RanBP3 (magenta). The GTP analog GppNHp is in navy blue. **B–D**. Details of the interface. The carbon atoms of RanBP2-1 and RanBP3 residues are shown in green and magenta, respectively. **B**. The Glu→Ser and Lys→Val substitutions within the ^1154^EWKER/^349^SWVER motif are predicted to disrupt two salt bridge interactions between Ran and the RBD. **C, D**. The R1284/S423 (C) and Q1248/G381 (D) substitutions are predicted to disrupt interactions with the Ran ^211^DEDDDL motif and with Glu158 in the G-domain, respectively. **E**. RBD surface plots. The two left-hand panels are related to (A) by an approximately 180° rotation about the vertical axis. *Top*, RanBP2-1 residues within 4 Å of Ran are coloured blue. *Bottom*, RanBP3 residues identical to RanBP2-1 are coloured magenta. The conserved and contact surfaces are similar in the vicinity of the Ran G-domain and linker (left panels), but not near the Ran C-helix (right panels).

Structures of a Ran-bound RBD have been determined in the context of three different complexes: the RanBP2-1/Ran, RanBP1/Ran/RanGAP and Yrb1/Ran/CRM1 complexes (Yrb1 is yeast RanBP1) [33], [38], [39]. The Ran-RBD interface is essentially identical in all these structures, as residues mediating Ran recognition are highly conserved across the three RBDs (Figure 3C) and because the interface is unaffected by either CRM1 or RanGAP binding. The Ran-RBD interaction has been described as a “molecular embrace” [38], in which Ran wraps its C-terminal tail around the RBD, and the RBD wraps its N-terminal extension around Ran (Figure 4A). The intermolecular contacts thus fall into three classes: those between the RBD N-terminal extension and the globular Ran guanine-nucleotide binding domain (G domain) (class 1); those between the Ran C-terminal tail and the RBD globular domain (class 2); and those between the two globular domains (class 3). Class 1 contacts are mediated by 8 residues in the RBD N-terminal extension, of which 5 make van der Waals contacts and 3 make H bonds via main chain atoms (Figure 3C). Class 2 contacts involve all three moieties of the Ran C-terminal tail (linker, C-helix, DEDDDL motif) and comprise: H bonds between the Ran linker and RBD strands β3 and β4, van der Waals contacts between the Ran C-helix and the RBD surface depression (asterisk in Figure 3B), and electrostatic interactions between the DEDDDL motif and basic RBD residues. Class 3 includes salt bridge interactions between the EWKER motif in RBD strand β2 and the nucleotide-binding effector loop of Ran.

To understand why RanBP3 binds RanGTP with such low affinity, we modelled a RanBP3-RBD/Ran complex by superimposing our RBD crystal structure onto that of the RanBP2-1/Ran complex and by altering the side chains of the RanBP2-1 N-terminal extension to match the RanBP3 sequence. Intermolecular contacts from all three classes are either absent or are partly compromised in this model. First, the HFEPVV motif within the N-terminal extension of high-affinity RBDs is poorly conserved in RanBP3, which lacks the three residues (underlined) that mediate hydrophobic contacts with Ran (Figure 3C). Second, substitutions within the EWKER motif (RanBP3 residues ^49^ SWVER) are predicted to disrupt salt bridge interactions with the Ran effector loop (Figure 4B). Third, the substitution of charged residues by Gly and Ser residues at positions 381 and 423 is predicted to disrupt hydrogen bonds with the Ran G-domain and DEDDDL motif, respectively (Figure 4C and D). Finally, four of the five RanBP2-1 residues that make van der Waals contacts with the Ran C-helix are not conserved in RanBP3 (Figure 3C). All together, nearly half of the intermolecular interactions observed in the Ran/RanBP2-1 complex are compromised in our Ran/RanBP3-RBD model, sufficiently accounting for the poor affinity observed.

Interestingly, the intermolecular contacts predicted to be conserved in our model are not distributed in a random manner. This is readily seen by comparing the RBD surface contacted by Ran with the surface conserved between RanBP3-RBD and RanBP2-1 (Figure 4E). Whereas the RBD surface regions that interact with the Ran G-domain and linker are well conserved, those that contact the C-helix are not. This, and the predicted loss of a H-bond to the DEDDDL-motif (Figure 4C), suggests that the Ran C-terminal tail may wrap less stably around the RanBP3 RBD, or that the path of the C-helix and DEDDDL motif along the RanBP3 RBD surface may differ significantly from that observed for the high-affinity RBDs. This is similar to a recent conjecture (based on differences in electrostatic potential of the RBD surfaces; Figure S5) that RanBP3 binds the DEDDDL motif more weakly than RanBP1 [33].

### Insights into cofactor activity

**Figure 5:**
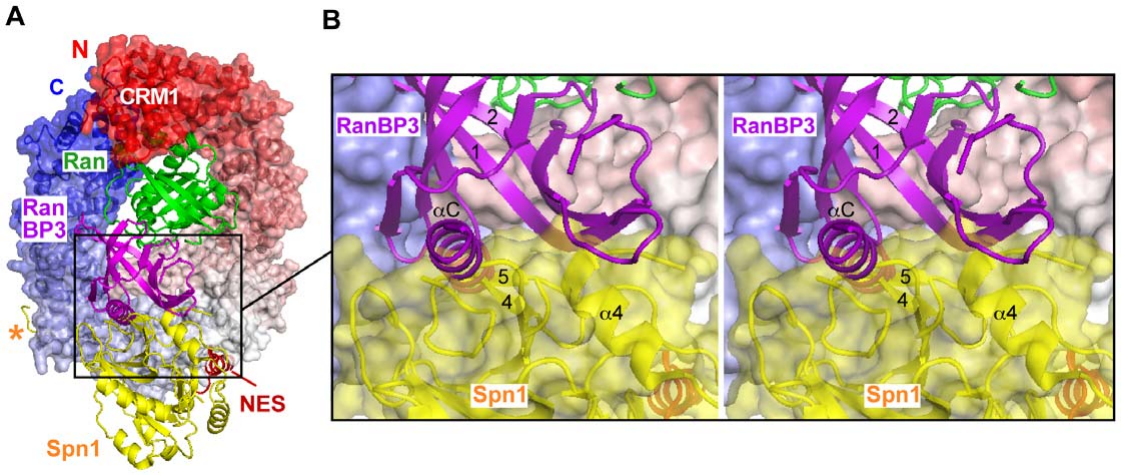
Steric clash between the RanBP3 RBD and Spn1. **A**. The Ran/RanBP3-RBD model was aligned with the CRM1/Ran/Spn1 crystal structure [15]. CRM1 is coloured from N- to C-terminus as a rainbow from red to white to blue. The nucleotide binding domain of Spn1 is in yellow, while the NES is in red; the asterisk indicates the C-terminal CRM1-binding epitope of Spn1. **B**. Stereoview of the region of steric overlap.

RanBP3 alters the cargo-binding selectivity of CRM1, favouring the binding of NES-bearing peptides over that of Spn1 [23]. How does this occur? Aligning our model of a RanBP3-RBD/Ran complex onto the CRM1/Ran/Spn1 structure reveals a severe steric clash between the RanBP3 RBD and Spn1 (Figure 5). The clash involves two regions of the nucleotide-binding domain of Spn1: helix α4, which overlaps with strand β1 of the RBD, and strands β4 and β5, which overlap with the RBD C-terminal helix. In contrast, neither the N-terminal NES nor the C-terminal CRM1-binding epitope of Spn1 are involved in the clash. This suggests that RanBP3 weakens the interaction with Spn1 by inhibiting the nucleotide-binding domain from stably associating with CRM1, leaving only the NES motif and C-terminal epitope to interact. The isolated NES of Spn1 is known to have lower affinity for CRM1 than canonical NESs such as those in PKI or Rev [17]. Hence, inhibiting the nucleotide-binding domain from interacting with CRM1 would allow canonical NES-bearing peptides to compete with Spn1 more effectively, explaining why RanBP3 increases the selectivity of CRM1 towards such cargos relative to Spn1 [23]. More generally, at a distance of ∼30 Å from the NES-binding groove, the RanBP3 RBD is well positioned to influence the cargo selectivity of CRM1 by interacting with (or excluding) cargo moieties that occupy volumes adjacent to (or overlapping with) that occupied by the RBD on the CRM1 surface.

**Figure 6:**
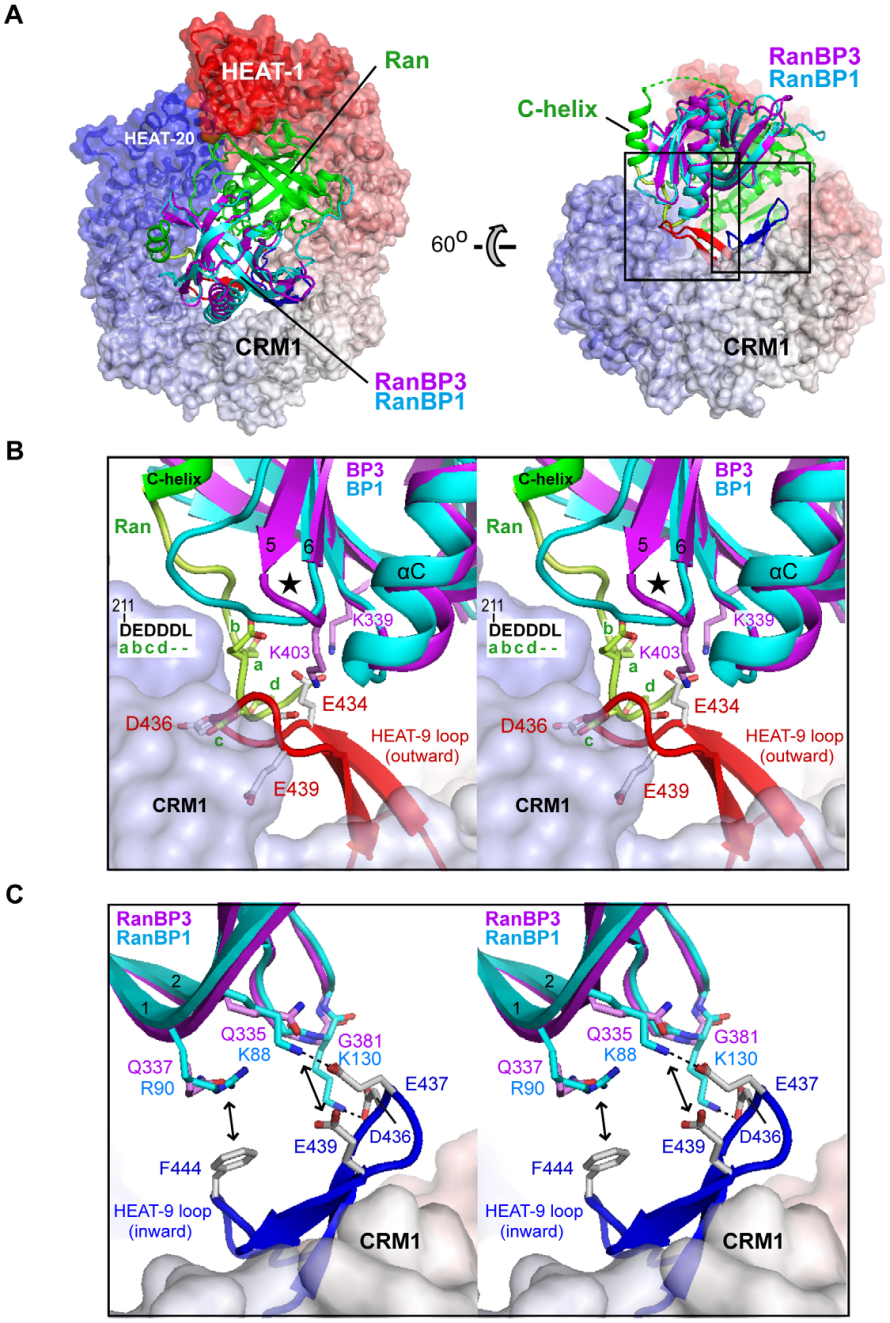
Structure of the CRM1/Ran/RanBP1 complex and alignment with RanBP3 RBD. **A**. Murine CRM1 from the CRM1/Ran/Spn1 complex [15] was aligned with yeast CRM1 from the CRM1/Ran/ RanBP1 complex [33] by superimposing the B helices of HEAT repeats 9–17; RanBP3 RBD was superimposed onto RanBP1. For clarity, only the HEAT-9 loop of murine CRM1 is shown (in red; that of yeast CRM1 is in blue). Yeast CRM1 is coloured from N- to C-terminus as a rainbow from red to white to blue. The large and small boxes indicate regions viewed in B and C, respectively. **B**. Close-up stereoview of the Ran C-terminal region. The DEDDDL motif of Ran (yellow) overlaps sterically with the outward conformation of the HEAT-9 loop (red). The black star indicates the β5β6 loop of the RBD, which is shorter in RanBP3 than in RanBP1. Favourable interactions are predicted between RanBP3 residues Lys339 and Lys403 and acidic residues in the HEAT-9 loop. For clarity, Ran residues 211–214 are labelled a–d and the G-domain is not shown. **C**. Close-up stereoview of the inward HEAT-9 loop conformation (blue). Double arrows indicate cation-pi and electrostatic interactions. In (B) and (C), CRM1 and Ran residues are labelled according to the human/murine numbering. (Human CRM1 residues 434–444 and Ran residues 211–214 correspond to yeast CRM1 residues 445–455 and Ran residues 213–216, respectively). RanBP1 residues follow the yeast numbering. All RanBP1 and CRM1 residues shown in (C) are conserved between yeast and human.

Unlike RanBP1 and RanBP2, which disassemble CRM1 export complexes, RanBP3 stabilizes the latter by increasing the affinity of CRM1 for RanGTP. To understand how this difference arises, we aligned the structures of the RanBP3 RBD and of CRM1 from the CRM1/Ran/Spn1 complex [15] onto the structure of the CRM1/Ran/RanBP1 complex [33] (Figure 6A). RanBP1 disassembles export complexes by causing the Ran-interacting HEAT-9 loop to switch from an “outward” conformation that promotes favourable interactions with Ran (red loop in Figure 6A) to an “inward” conformation (blue loop) that induces a constriction of the NES-binding groove and consequent NES release [33]. RanBP1 accomplishes this by both destabilizing the outward and stabilizing the inward conformations. It destabilizes the outward conformation by positioning the Ran C-terminal DEDDDL motif so as to cause steric and electrostatic repulsion with the HEAT-9 loop (Figure 6B). The protrusion on the RanBP1 surface formed by the β5β6 loop plays an important role here, as it guides the DEDDDL motif into position. In contrast, this protrusion is missing from the RanBP3 RBD (Figure S4B), which, as discussed above, likely binds the Ran C-helix and DEDDDL motif more weakly than RanBP1 and/or positions these elements differently.

Conversely, RanBP1 stabilizes the HEAT-9 loop in its inward conformation through interactions mediated by three basic residues located in RanBP1 strand 1 and in the β3β4 loop. RanBP1 residues Lys88 and Lys130 make salt bridge interactions with three acidic residues in the HEAT-9 loop (Asp436, Glu437 and Glu439), while RanBP1 residue Arg90 participates in a cation-pi interaction with loop residue Phe444 (Figure 6C). None of these interactions would be conserved in a RanBP3/CRM1 complex, as the three basic RanBP1 residues are replaced in RanBP3 by a glycine and two glutamine residues. In short, RanBP1 destabilizes the outward HEAT-9 loop conformation and favours the inward conformation, whereas the RanBP3 RBD appears poorly suited for stabilizing the inward, but compatible with the outward, conformation. These differences rationalize why RanBP1 disassembles CRM1 export complexes whereas RanBP3 does not.

How then does RanBP3 increase the affinity of CRM1 towards Ran? Our structural alignment suggests that the RanBP3 RBD could feasibly stabilize the HEAT-9 loop in its outward conformation, as residues in strand 1 and in the β5β6 turn are well placed to interact favourably with residues in the HEAT-9 loop. These include RanBP3 residues Lys339 and Lys403, which could potentially form salt bridge interactions with CRM1 residue Glu434 (Figure 6B). As has been previously noted [42], stabilizing the outward HEAT-9 loop conformation is an obvious way by which RanBP3 could increase the affinity of CRM1 for Ran. Admittedly, however, such a role for the RBD would provide at best only a partial explanation of how RanBP3 functions, as it ignores the contribution of the FxFG-containing region known to mediate CRM1 binding and to be crucial for cofactor activity [24]. It is tempting to speculate that the binding of this region to CRM1 destabilizes the inward conformation of the HEAT-9 loop, allowing for the latter to be subsequently stabilized in its outward conformation by the RBD. However, fully elucidating the mechanism by which RanBP3 increases the Ran-binding affinity of CRM1 will require further study, including the structure determination of a CRM1/RanBP3 complex.

### Concluding remarks

The five RBDs of RanBP1 and RanBP2 are phylogenetically closely related, whereas the RBD of RanBP3 diverges considerably from these. One might therefore reasonably expect a detailed comparison of the divergent RBD structures to yield a better understanding of the strikingly different roles performed by the corresponding proteins in CRM1-mediated export. Indeed, the structural analysis presented above offers highly plausible explanations for why RanBP3 binds Ran more weakly than RanBP1 and RanBP2, how RanBP3 modulates the cargo selectivity of CRM1, and why RanBP3 fails to disassemble export complexes. In addition, it provides clues into how RanBP3 increases the Ran-binding affinity of CRM1 in the nucleus. Fully unravelling this last question remains an important challenge for future study.

## Materials and Methods

### Protein expression and purification

Human full-length RanBP3 (residues 1–499; isoform B) and two RanBP3 RBD constructs (residues 310–454 for ITC and residues 320–454 for crystallization) were cloned in a pETM11 vector as fusion constructs containing an N-terminal His tag and a TEV protease cleavage site. Transformed *E. coli* strain BL21 (DE3) Gold (Stratagene) cells were grown in LB medium containing kanamycin (50 µg/mL) until reaching an OD_600_ of 0.8, induced with 0.5 mM isopropyl β-D-thiogalactoside (IPTG) and incubated for a further 3 h at 37° before harvesting. Cells were lysed by sonication in 20 mM Tris pH 8, 200 mM NaCl, 5 mM imidazole, 1 mM β-mercaptoethanol, 1 mM PMSF and Complete protease inhibitor EDTA-free (1 tablet/50 ml; Roche). The clarified lysate was incubated with Ni-NTA resin (Qiagen) and washed with buffer A (20 mM Tris pH 8, 150 mM NaCl, 10 mM imidazole, 1 mM β-mercaptoethanol, 1 mM PMSF). Proteins were eluted with 250 mM imidazole, dialysed overnight in the presence of His-tagged TEV protease against buffer A containing no imidazole, and incubated with Ni-NTA resin to remove His-tag containing species. Proteins were further purified on a Superdex 75 column (GE Healthcare) in 20 mM Tris pH 8, 100 mM NaCl and 1 mM DTT and concentrated on a Centricon centrifugal filtration device (Millipore).

Full-length human Ran containing the Q69L mutation was expressed from a pPROEx vector as an N-terminally His-tagged protein containing a TEV protease site. Transformed *E. coli* BL21 (DE3) CodonPlus RILP (Stratagene) cells were grown in TB medium containing ampicillin (100 µg/ml) and chloramphenicol (30 µg/ml) until reaching an OD_600_ of 0.8, induced with 0.5 mM IPTG and incubated overnight at 18°C before harvesting. Cells were lysed in buffer B (30 mM Tris pH 7.4, 180 mM NaCl, 10 mM imidazole pH 7.4, 5 mM MgCl_2_, 2 mM β-mercaptoethanol, Complete protease inhibitor EDTA-free (1 tablet/50 mL; Roche)) containing DNase I (10 µg/mL) and lysozyme (1 mg/mL) using a French Press. The clarified lysate was loaded onto a HisTrap column (GE Healthcare) and Ran was eluted with an imidazole gradient (0–400 mM). The protein was dialysed overnight in the presence of His-tagged TEV protease against buffer B containing 0 mM imidazole plus 10% glycerol, and subsequently passed over a second HisTrap column to remove His-tag containing species. Ran was then applied to a MonoQ HR 5/5 column (GE Healthcare) pre-equilibrated in 50 mM Tris pH 7.4, 5 mM MgCl and 2 mM DTT, and purified protein was recovered in the flow-through. The protein was concentrated by using a HiTrap SP (GE Healthcare) column pre-equilibrated in 20 mM Tris pH 7.4, 5 mM MgCl_2_, 5 mM DTT and eluting with a NaCl gradient (0–1.5 M). RanQ69L was loaded with GTP by incubating with a 300-fold molar excess of GTP in the presence of 10 mM EDTA at 4°C for 1 hour and then at 20°C for an additional 1–2 hours, followed by addition of 15 mM MgCl_2_ to halt the nucleotide exchange reaction. The protein was separated from unbound nucleotides by Superdex 75 (GE Healthcare) chromatography in 10 mM Tris pH 7.5, 70 mM NaCl, 5 mM MgCl_2_ and 1 mM DTE. HPLC analysis confirmed that RanQ69L was fully charged with GTP.

### Isothermal Titration Calorimetry

ITC was carried out at 25°C using a VP-ITC Microcal calorimeter (Microcal, Northhampton, MA, USA). All proteins were freshly purified and dialysed extensively against ITC buffer (300 mM NaCl, 5 mM MgCl_2_, 10 mM TRIS pH 7.4) prior to ITC measurements. Titrations consisted of 6–12 µL aliquot injections made at time intervals of 5 min to ensure that the titration peak returned to the baseline. The ITC data were analyzed and corrected for the heat of dilution of injectant into buffer using program Origin version 5.0 provided by the manufacturer.

### Structure determination

The RanBP3 RBD was crystallized by hanging drop vapour diffusion at 20°C by mixing 12 mg/mL (crystal form 1) or 20 mg/mL (crystal form 2) protein with an equal volume of crystallization buffer. Crystal form 1 grew from 1.5 M Li_2_SO_4_, 100 mM Hepes pH 7.5, and form 2 grew from 30% PEG 3350, 50 mM TRIS pH 8.5. All crystals were cryo-protected in 30% glycerol. Diffraction data were processed using XDS [43] or MOSFLM [44] and programs of the CCP4 suite [45]. The structure of crystal form 1 was determined by the SIRAS method, as attempts to solve the structure by molecular replacement using the available NMR structure (PDB id 2CRF) failed. Diffraction data were collected at 2 Å resolution from a crystal soaked for 3 hours in 2 mM K_2_PtCl_4_ and at 1.6 Å resolution from a native crystal on ESRF beamline ID23-1 (λ = 1.0723 Å). Three Pt sites were located and phases calculated using programs SHELXD and SHELXE [46]. The resulting electron density map was almost entirely autotraced by ARP/wARP [47], with the remainder traced manually in COOT [48]. The final structure was refined against the native data (R_cryst_ = 14.7%, R_free_ = 19.6%) using program Phenix [49]. (Hydrogen atoms were not included in the refinement). Diffraction data from crystal form 2 were collected at 2.1 Å resolution at ESRF beamline ID14-4 (λ = 0.9795 Å). The structure was solved by molecular replacement using program PHASER [50] and refined to an R-value of 19.3% (R_free_ = 22.5%) and good geometry (Table 1). Structures were determined for both the wildtype protein and a double point mutant (E352A/R352V) compromised for Ran binding [23]. These structures are identical except for the side chains at the mutated positions. Figures were made with Pymol (DeLano scientific LLC, San Carlos, CA, USA, http://www.pymol.org).

### Accession numbers

Coordinates and structure factors for the wildtype and E352A/R352V mutant of the RanBP3 RBD have been deposited in the Protein Data Bank (PDB) under the accession codes 2Y8F and 2Y8G, respectively.

## Supporting Information

Figure S1 **ITC profile of Ran binding by full-length RanBP3.** Differential power (ΔP) time course of raw injection heats for a titration of 530 µM RanQ69L∶GTP into 48 µM RanBP3. The inset shows normalized binding enthalpies corrected for the heat of dilution as a function of binding site saturation. The solid line represents a nonlinear least squares fit using a single-site binding model. K_d,obs_ was 15±3 µM and the stoichiometry was 0.78±0.06. The shape of the curve suggests the presence of additional processes (not observed with the isolated RBD; Figure 2) having different kinetics than the dilution and binding reactions. This conceivably may be due to residues within the intrinsically disordered N-terminal domain of RanBP3 changing conformation in the presence of Ran.
(TIF)

Figure S2 **Alignment of RanBP3 RBD molecules.** The two molecules (1A and B) in the asymmetric unit of crystal form 1 and the four (2A–D) from crystal form 2 were structurally aligned and are shown as a Cα trace in stereoview. The β2β3 loop is disordered in molecules 2B, 2C and 2D, while the β6β7 loop is disordered in molecules 1A and 1B. The N-terminal 10 residues are disordered in all molecules except 2A and 2D. In molecule 2A, these residues fold back to pack loosely against strands β2 and β3; in molecule 2D, the N-terminal residues include a small α helix (res. 322–327; asterisk) and extend outward to interact with 3 neighbouring molecules in the crystal lattice.
(TIF)

Figure S3 **Alignment of RBD structures shown as a stereo Cα trace.** The structures of RanBP1 [39], RanBP2-1 [38], RanBP2-2 [40] and Yrb1 [33] (corresponding to PDB entires 1K5G, 1RRP, 1XKE and 3M1I, respectively) were aligned onto the RanBP3 RBD structure.
(TIF)

Figure S4 **Structural differences in the β5β6 loop.** **A**. Structural alignment of RanBP3-RBD (magenta) with RanBP1 (green). The view is that of Figure 2B, right panel. *Inset:* Stereoview of the β5β6 loop. Residues in RanBP1 and RanBP3 are shown with carbon atoms coloured green and magenta, respectively. **B**. Surface representation of the RBD from RanBP3 and RanBP1 and of the second RBD of RanBP2. The asterisk indicates the surface depression that recognizes the Ran C-helix, which is markedly more pronounced in RanBP1 and RanBP2-1 than in the RanBP3 RBD. The surface corresponding to the β5β6 loop is coloured more darkly.
(TIF)

Figure S5 **Comparison of RBD surfaces.** **A**. Ribbon diagram of the RanBP1/Ran complex [39]. Side chains are shown for acidic residues within the C-terminal ^211^DEDDDL motif of Ran. **B**. Electrostatic surface plots of RanBP1, RanBP2-1 [38] and the RanBP3 RBD, with the Ran C-terminal tail from the RanBP1/Ran complex superposed to facilitate comparison. The regions indicated by an oval show that the RanBP3 RBD has a distinctly less basic character in the vicinity of the DEDDDL motif than the other two RBDs, as previously pointed out [33]. The figure was prepared using program CCP4MG and is coloured from −0.5 V (red) to +0.5 V (blue).
(TIF)

Table S1 **Comparison of RanBP3 RBD molecules in the two crystal forms.** * Rmsd values below the diagonal are for pairwise alignments made using all Cα atoms; subscripts indicate the number of Cα atoms aligned (these vary between 108 and 121 because the residues that are disordered vary among molecules). Values above the diagonal are for alignments made using a common core of 93 Cα atoms (residues 330–343, 350–363, 373–409, 418–446; i.e. excluding the N- and C-termini and variable loops). † rmsd_100_ is the normalized rmsd value of Carugo and Pongor [42], which allows one to compare two or more rmsd values calculated from alignments made using different numbers of Cα residues: rmsd_100_ = rmsd/{1+ln [ (N/100)^½^ ]}. Pairwise alignments of the six structures yield a mean rmsd_100_ value of 1.51 Å, primarily reflecting variations in the N- and C-terminal regions and in the β1β2, β2β3 and β6β7 loops. Excluding these regions yields a much lower value (0.64 Å), indicating that the core structure is highly conserved.
(DOC)

Table S2 **Comparison of RBD structures.** † Structures were aligned against RanBP3 residues 330–446 and rmsd values were calculated for all equivalent (between 97 and 116) Cα positions. Pairwise alignments were made between all six molecules of the RanBP3-RBD structure (1A–B, 2A–2D) and all chains in the X-ray structures (1K5G: 4 chains, 1RRP 2 chains, 3MI1, 1 chain); for NMR structure 1XKE the 1^st^, 10^th^, and 20^th^ models in the PDB file were used. Values were then converted to rmsd_100_ scores (see Table S1) and averaged. * Pairwise structural alignments involving RanBP1, RanBP2-1, RanBP2-2 and Yrb1 (multiple chains for each structure) yielded between 99 and 134 topologically equivalent Cα positions. The resulting rmsd values were converted to rmsd_100_ scores and averaged. Alignments involving RanBP2-2 systematically give higher rmsd values because of greater coordinate errors in the NMR model compared to the high-resolution crystal structures.
(DOC)
