# Supplementary figures and images for: Insights into the Function of the CRM1 Cofactor RanBP3 from the Structure of Its Ran-Binding Domain
Source: PLoS One. 2011 Feb 25;6(2):e17011. doi: 10.1371/journal.pone.0017011 (PMC3045386; doi:10.1371/journal.pone.0017011)

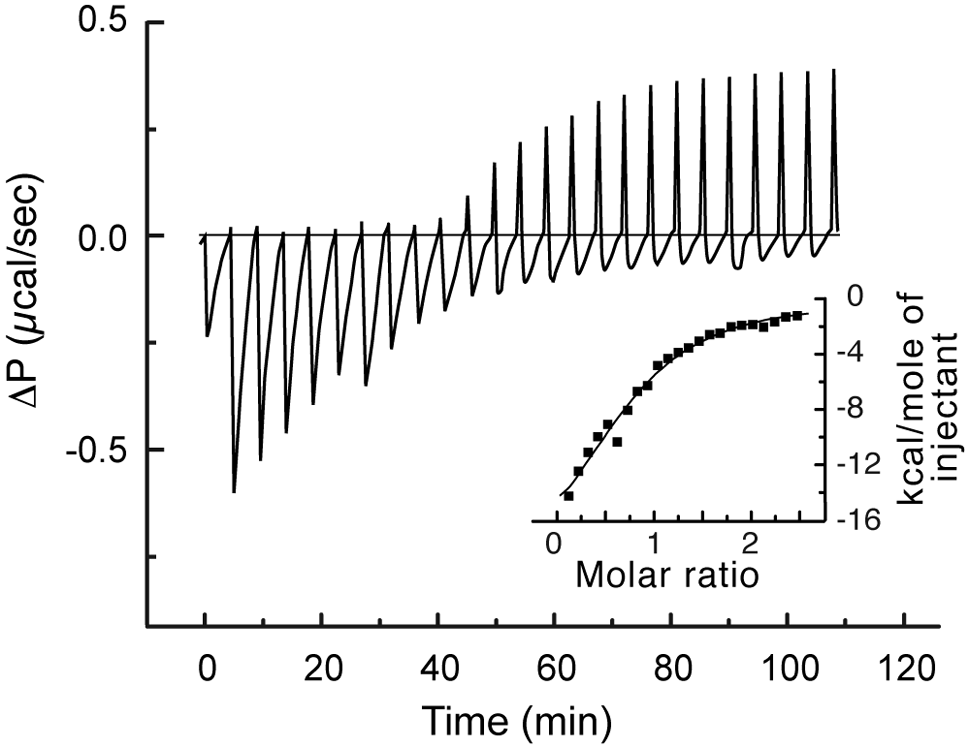

Supplement: Figure S1 — ITC profile of Ran binding by full-length RanBP3. Differential power (ΔP) time course of raw injection heats for a titration of 530 µM RanQ69L∶GTP into 48 µM RanBP3. The inset shows normalized binding enthalpies corrected for the heat of dilution as a function of binding site saturation. The solid line represents a nonlinear least squares fit using a single-site binding model. Kd,obs was 15±3 µM and the stoichiometry was 0.78±0.06. The shape of the curve suggests the presence of additional processes (not observed with the isolated RBD; Figure 2) having different kinetics than the dilution and binding reactions. This conceivably may be due to residues within the intrinsically disordered N-terminal domain of RanBP3 changing conformation in the presence of Ran. (TIF) [file pone.0017011.s001.tif]

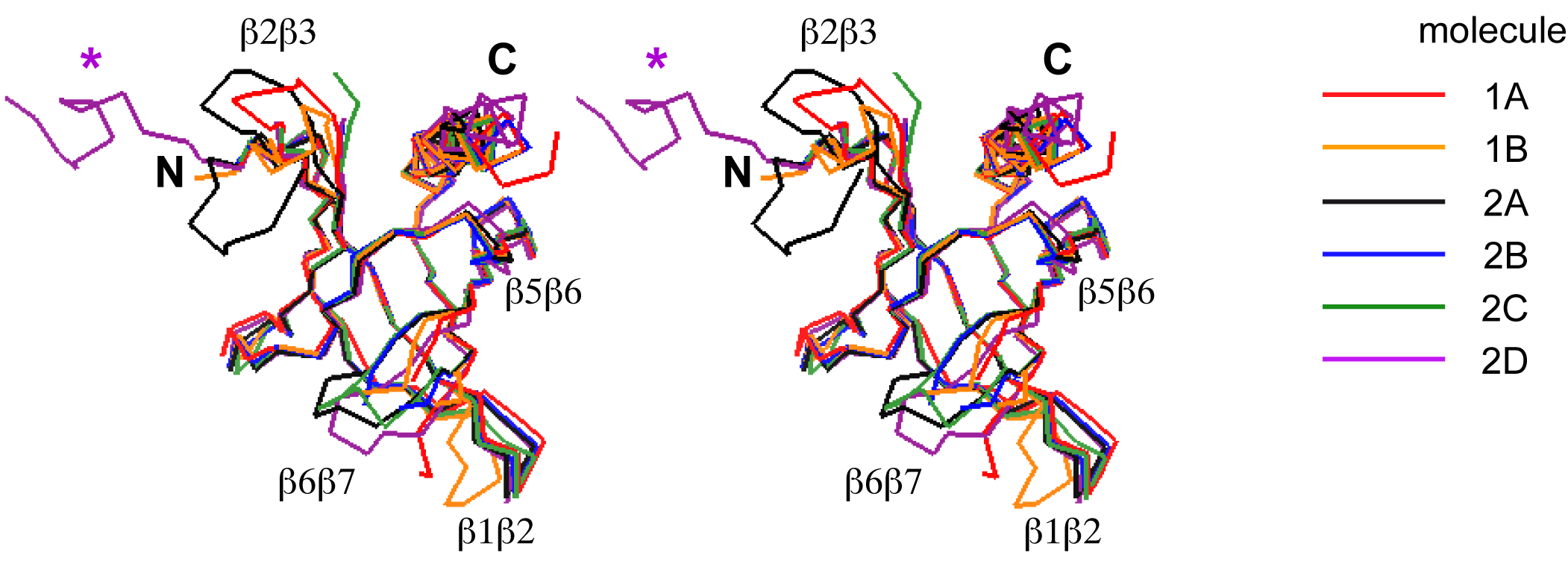

Supplement: Figure S2 — Alignment of RanBP3 RBD molecules. The two molecules (1A and B) in the asymmetric unit of crystal form 1 and the four (2A–D) from crystal form 2 were structurally aligned and are shown as a Cα trace in stereoview. The β2β3 loop is disordered in molecules 2B, 2C and 2D, while the β6β7 loop is disordered in molecules 1A and 1B. The N-terminal 10 residues are disordered in all molecules except 2A and 2D. In molecule 2A, these residues fold back to pack loosely against strands β2 and β3; in molecule 2D, the N-terminal residues include a small α helix (res. 322–327; asterisk) and extend outward to interact with 3 neighbouring molecules in the crystal lattice. (TIF) [file pone.0017011.s002.tif]

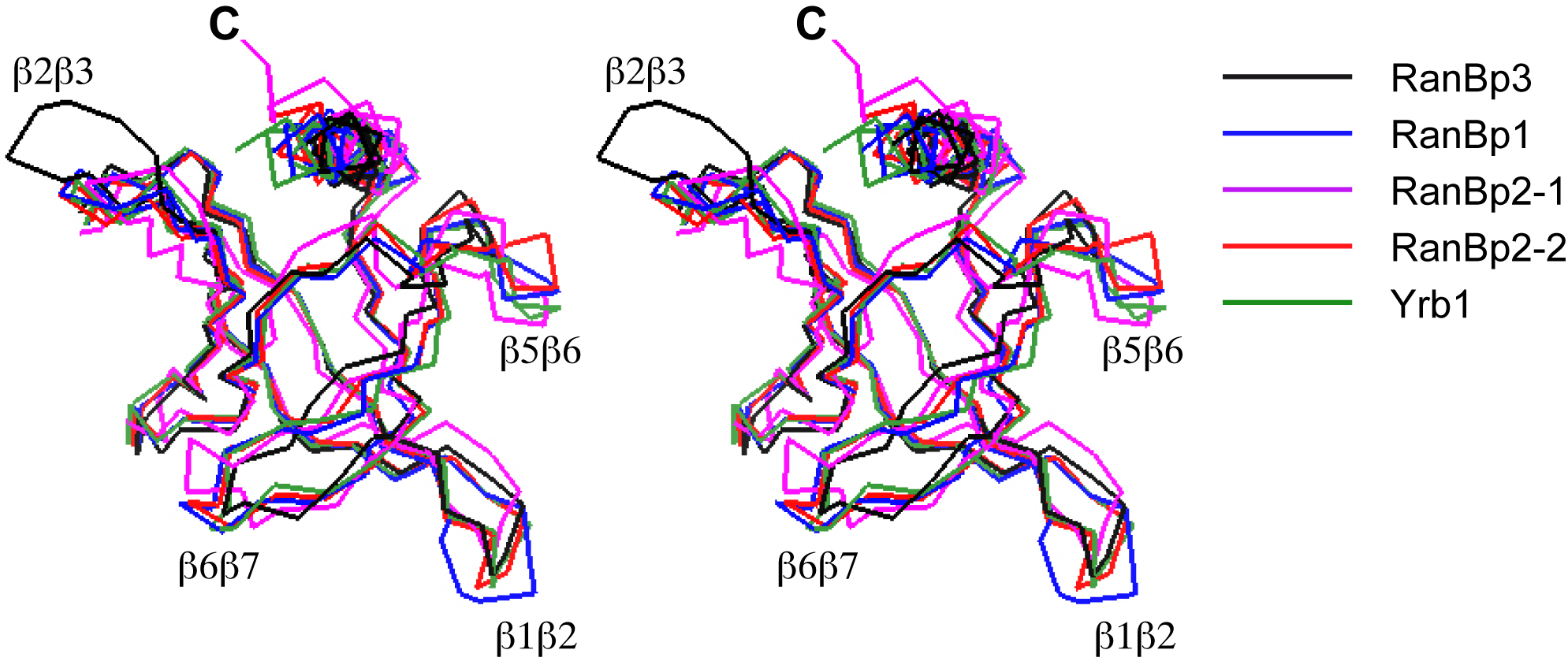

Supplement: Figure S3 — Alignment of RBD structures shown as a stereo Cα trace. The structures of RanBP1 [39], RanBP2-1 [38], RanBP2-2 [40] and Yrb1 [33] (corresponding to PDB entires 1K5G, 1RRP, 1XKE and 3M1I, respectively) were aligned onto the RanBP3 RBD structure. (TIF) [file pone.0017011.s003.tif]

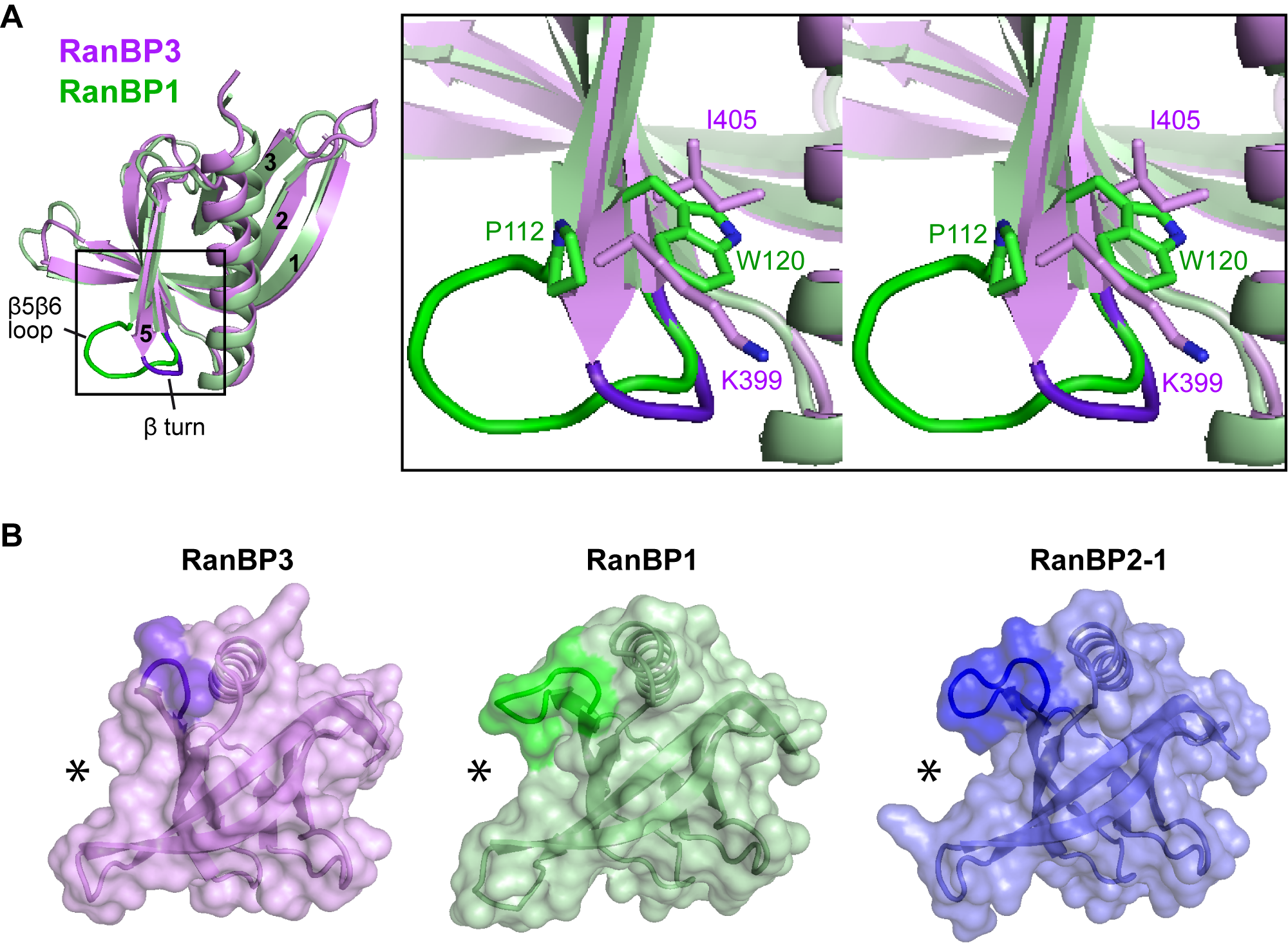

Supplement: Figure S4 — Structural differences in the β5β6 loop. A. Structural alignment of RanBP3-RBD (magenta) with RanBP1 (green). The view is that of Figure 2B, right panel. Inset: Stereoview of the β5β6 loop. Residues in RanBP1 and RanBP3 are shown with carbon atoms coloured green and magenta, respectively. B. Surface representation of the RBD from RanBP3 and RanBP1 and of the second RBD of RanBP2. The asterisk indicates the surface depression that recognizes the Ran C-helix, which is markedly more pronounced in RanBP1 and RanBP2-1 than in the RanBP3 RBD. The surface corresponding to the β5β6 loop is coloured more darkly. (TIF) [file pone.0017011.s004.tif]

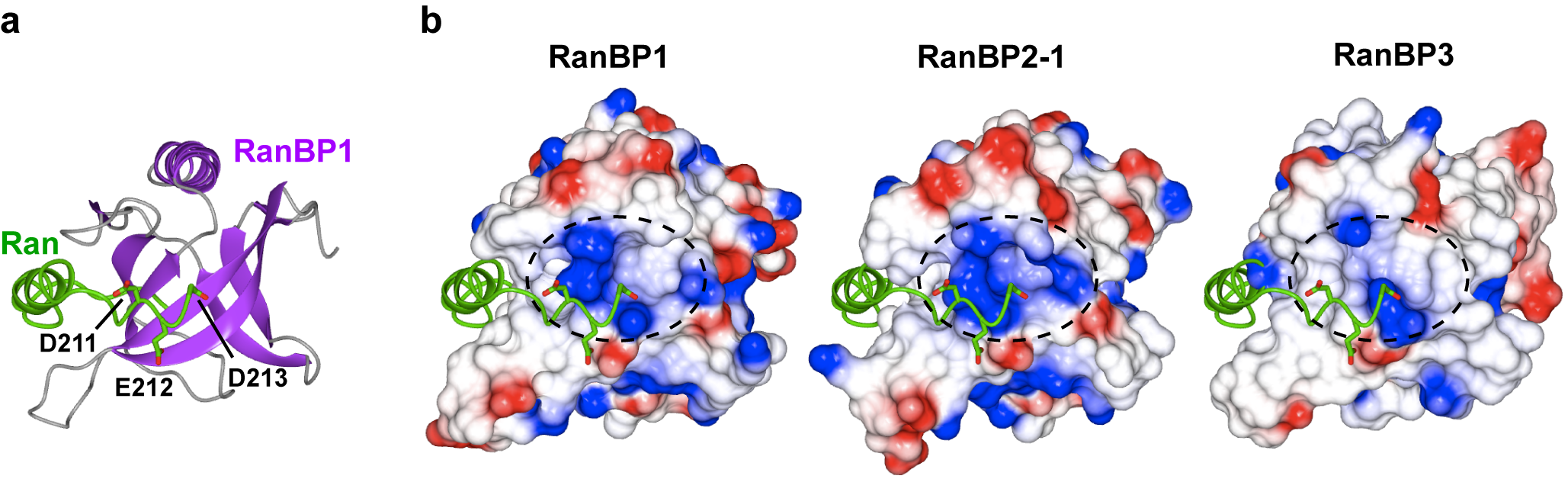

Supplement: Figure S5 — Comparison of RBD surfaces. A. Ribbon diagram of the RanBP1/Ran complex [39]. Side chains are shown for acidic residues within the C-terminal 211DEDDDL motif of Ran. B. Electrostatic surface plots of RanBP1, RanBP2-1 [38] and the RanBP3 RBD, with the Ran C-terminal tail from the RanBP1/Ran complex superposed to facilitate comparison. The regions indicated by an oval show that the RanBP3 RBD has a distinctly less basic character in the vicinity of the DEDDDL motif than the other two RBDs, as previously pointed out [33]. The figure was prepared using program CCP4MG and is coloured from −0.5 V (red) to +0.5 V (blue). (TIF) [file pone.0017011.s005.tif]
