# Supplementary material for: Insights into the Function of the CRM1 Cofactor RanBP3 from the Structure of Its Ran-Binding Domain
Source: PLoS One. 2011 Feb 25;6(2):e17011. doi: 10.1371/journal.pone.0017011 (PMC3045386; doi:10.1371/journal.pone.0017011)
Supplement: Table S1 — Comparison of RanBP3 RBD molecules in the two crystal forms. * Rmsd values below the diagonal are for pairwise alignments made using all Cα atoms; subscripts indicate the number of Cα atoms aligned (these vary between 108 and 121 because the residues that are disordered vary among molecules). Values above the diagonal are for alignments made using a common core of 93 Cα atoms (residues 330–343, 350–363, 373–409, 418–446; i.e. excluding the N- and C-termini and variable loops). † rmsd100 is the normalized rmsd value of Carugo and Pongor [42], which allows one to compare two or more rmsd values calculated from alignments made using different numbers of Cα residues: rmsd100 = rmsd/{1+ln [ (N/100)½ ]}. Pairwise alignments of the six structures yield a mean rmsd100 value of 1.51 Å, primarily reflecting variations in the N- and C-terminal regions and in the β1β2, β2β3 and β6β7 loops. Excluding these regions yields a much lower value (0.64 Å), indicating that the core structure is highly conserved. (DOC) [file pone.0017011.s006.doc]

**Table S1.** **Comparison of RanBP3 RBD molecules in the two crystal forms.**

| **^*^rmsd (Å)** | | | | | | |
| --- | --- | --- | --- | --- | --- | --- |
| **Molecule** | **1A** | **1B** | **2A** | **2B** | **2C** | **2D** |
| 1A | -- | 0.71 | 0.57 | 0.65 | 0.77 | 0.80 |
| 1B | 1.99_116_ | -- | 0.48 | 0.47 | 0.47 | 0.74 |
| 2A | 1.87_116_ | 2.21_117_ | -- | 0.53 | 0.57 | 0.67 |
| 2B | 1.42_110_ | 0.83_109_ | 1.31_108_ | -- | 0.42 | 0.57 |
| 2C | 1.64_112_ | 1.38_112_ | 1.17_113_ | 0.70_108_ | -- | 0.78 |
| 2D | 1.65_117_ | 1.59_113_ | 4.06_121_ | 1.08_111_ | 1.33_113_ | -- |
| ^†^mean rmsd_100_ | overall | | | core | | |
|  | 1.51 ± 0.71 | | | 0.64 ± 0.13 | | |

* Rmsd values below the diagonal are for pairwise alignments made using all Cα atoms; subscripts indicate the number of Cα atoms aligned (these vary between 108 and 121 because the residues that are disordered vary among molecules). Values above the diagonal are for alignments made using a common core of 93 Cα atoms (residues 330-343, 350-363, 373-409, 418-446; i.e. excluding the N- and C-termini and variable loops).

† rmsd_100_ is the normalized rmsd value of Carugo and Pongor [42], which allows one to compare two or more rmsd values calculated from alignments made using different numbers of Cα residues: rmsd_100_= rmsd / {1 + ln [ (N/100)^½^ ]}. Pairwise alignments of the six structures yield a mean rmsd_100_ value of 1.51 Å, primarily reflecting variations in the N- and C-terminal regions and in the β1β2, β2β3 and β6β7 loops. Excluding these regions yields a much lower value (0.64Å), indicating that the core structure is highly conserved.
