# Supplementary material for: Insights into the Function of the CRM1 Cofactor RanBP3 from the Structure of Its Ran-Binding Domain
Source: PLoS One. 2011 Feb 25;6(2):e17011. doi: 10.1371/journal.pone.0017011 (PMC3045386; doi:10.1371/journal.pone.0017011)
Supplement: Table S2 — Comparison of RBD structures. † Structures were aligned against RanBP3 residues 330–446 and rmsd values were calculated for all equivalent (between 97 and 116) Cα positions. Pairwise alignments were made between all six molecules of the RanBP3-RBD structure (1A–B, 2A–2D) and all chains in the X-ray structures (1K5G: 4 chains, 1RRP 2 chains, 3MI1, 1 chain); for NMR structure 1XKE the 1st, 10th, and 20th models in the PDB file were used. Values were then converted to rmsd100 scores (see Table S1) and averaged. * Pairwise structural alignments involving RanBP1, RanBP2-1, RanBP2-2 and Yrb1 (multiple chains for each structure) yielded between 99 and 134 topologically equivalent Cα positions. The resulting rmsd values were converted to rmsd100 scores and averaged. Alignments involving RanBP2-2 systematically give higher rmsd values because of greater coordinate errors in the NMR model compared to the high-resolution crystal structures. (DOC) [file pone.0017011.s007.doc]

Table S2. Comparison of RBD structures.

|  |  |  |  | **Mean rmsd_100_ (Å) / Sequence identity** | | | | |
| --- | --- | --- | --- | --- | --- | --- | --- | --- |
| **RBD**  **structure** | **Complex with** | **PDB ID** | **Method** | ^†^**RanBP3** | **^*^RanBP1** | **RanBP2-1** | **RanBP2-2** | **Yrb1** |
| RanBP3 | --- |  | X-ray | --- | 22% | 23% | 24% | 18% |
| RanBP1 | Ran, RanGAP | 1K5G | X-ray | 1.63 ± 0.20 | --- | 62% | 51% | 56% |
| RanBP2-1 | Ran | 1RRP | X-ray | 1.72 ± 0.21 | 0.89 ± 0.08 | --- | 56% | 52% |
| RanBP2-2 | --- | 1XKE | NMR | 2.12 ± 0.3 | 1.65 ± 0.04 | 1.57 ± 0.07 | --- | 47% |
| Yrb1 | Ran, CRM1 | 3M1I | X-ray | 1.90 ± 0.19 | 0.97 ± 0.01 | 0.86 ± 0.03 | 1.59 ± 0.05 | --- |

† Structures were aligned against RanBP3 residues 330-446 and rmsd values were calculated for all equivalent (between 97 and 116) Cα positions. Pairwise alignments were made between all six molecules of the RanBP3-RBD structure (1A-B, 2A-2D) and all chains in the X-ray structures (1K5G: 4 chains, 1RRP 2 chains, 3MI1, 1 chain); for NMR structure 1XKE the 1^st^, 10^th^, and 20^th^ models in the PDB file were used.  Values were then converted to rmsd_100_  scores (see Table S1) and averaged.

* Pairwise structural alignments involving RanBP1, RanBP2-1, RanBP2-2 and Yrb1 (multiple chains for each structure) yielded between 99 and 134 topologically equivalent Cα positions. The resulting rmsd values were converted to rmsd_100_ scores and averaged. Alignments involving RanBP2-2 systematically give higher rmsd values because of greater coordinate errors in the NMR model compared to the high-resolution crystal structures.
